# The MANTRA study: a new umbrella concept prospectively applied to assess implantable medical devices for heart valve procedures

**DOI:** 10.1186/s13019-023-02270-w

**Published:** 2023-04-07

**Authors:** Bart Meuris, Serdar Günaydın, Patrizio Lancellotti, Luigi Badano, Gabriel Aldea, Rita Herrenknecht, Elisa Cerutti, Sara Gaggianesi, Silvia Dipinto, Paola Morando, Jörg Kempfert

**Affiliations:** 1 University Hospital Leuven, Gasthuisberg Herestraat 49, 3000 Leuven, Belgium; 2 Ankara City Hospital Üniversiteler Mahallesi, 1604 Cadde No: 9, Çankaya, Ankara Turkey; 3 University Hospital Liège, CHU Sart Tilman, Avenue de l’hôpital, 1, 4000 Liège, Belgium; 4 Department of cardiology, Istituto Auxologico Italiano, IRCCS, Piazzale Brescia, 20, 20149 Milan, Italy; 5 Division of Cardiothoracic Surgery, University of Washington, Seattle, WA USA; 6 Corcym S.R.L, Via Giovanni Spadolini 7, 20141 Milan, Italy; 7 Klinik für Herz-, Thorax- und Gefäßchirurgie Deutsches Herzzentrum der Charité, Augustenburger Platz 1, 13353 Berlin, Germany; 8 Department of medicine and surgery, University of Milano-Bicocca, Piazza Ateneo Nuovo 1, 20126 Milan, Italy

**Keywords:** Study design, Medical devices, Aortic valve, Mitral valve, And tricuspid heart valve

## Abstract

**Background:**

Clinical evidence is commonly obtained through individual trials that are time-, cost- and resource-consuming, and which often leave unanswered clinically relevant questions. Umbrella studies have been developed to address the need for more efficient and flexible trial structures, predominantly for cancer treatments. The umbrella concept foresees data collection within a unifying trial structure, to which one or more substudies may be added at any time to address product- or therapy-specific questions. To our knowledge, the umbrella concept has not yet been used in the medical device area, but it may offer similar advantages as in other settings, particularly in areas where multiple therapies are available within one large treatment area.

**Methods:**

The MANTRA study (NCT05002543) is a prospective, global, post-marketing clinical follow-up study. The aim is to collect safety and device performance data covering the Corcym cardiac surgery portfolio for the treatment of aortic, mitral, and tricuspid valve diseases. The study uses a master protocol that outlines the main common parameters, and the specific questions are addressed in three substudies. The primary endpoints are device success at 30 days. Secondary endpoints include safety- and device performance-related data at 30 days, 1 year, and then annually through to 10 years. All endpoints are defined according to the more recent guidelines for heart valve procedures. Additionally, procedure and hospitalization information are collected, including Enhanced Recovery after Surgery in sites using such protocols, and patient outcome measures such as New York Heart Association classification and quality-of-life questionnaires.

**Results:**

The study started in June 2021. Enrollment in all three substudies is ongoing.

**Conclusions:**

The MANTRA study will provide contemporary information on the long-term outcomes of medical devices for the treatment of aortic, mitral, and tricuspid heart valve diseases in routine clinical practice. The umbrella approach adopted in the study has the potential of longitudinally assessing long-term efficacy of the devices and the flexibility to investigate new research questions as they arise.

## Background

Traditionally, clinical investigations in the medical device area have been conducted using individual study protocols designed for specific devices or device groups. The aim of such studies is to investigate safety and performance according to predefined endpoints and corresponding statistical analysis plans (SAPs). Any change to the study design and scope of the investigation mandated protocol amendments. Device improvements, iterations, or expansion of the clinical indications require new pre- or post-marketing studies, delaying product launch and requiring more resources from the manufacturers. In addition, regulatory requirements have evolved over time, and ad-hoc analyses are now frequently requested. Therefore, clinical scientists have been exploring novel study designs to increase flexibility without necessarily increasing time and costs of these studies. New ways to conduct clinical investigations, to collect data in one common platform, and to perform complex analyses have been explored in the pharmacological area for several years. In this context, adaptive investigational pathways have been adopted to address several questions simultaneously. Master protocols have been used in various contexts, outlined in Table 1 [1], whereby an overarching (“umbrella”) protocol exits under which multiple parallel studies are conducted. According to Park et al. [2], no umbrella trials have been conducted outside the field of oncology.

**Table 1:** Master protocol trials and corresponding objectives, according to Woodcock and LaVange, 2017 [1]

Trial type	Objective
Umbrella	Multiple targeted therapies in the context of a single disease
Basket	A single targeted therapy in the context of multiple diseases or disease subtypes
Platform	Multiple targeted therapies in the context of a single disease in a perpetual manner, with therapies allowed to enter or leave the platform on the basis of a decision algorithm

Although umbrella studies have been conducted in pharma trials, to our knowledge this is the first such study to apply this concept in the medical device field.

Even though the concept of an umbrella protocol is new to the medical device area, the main advantages are equally as applicable, in particular for device manufacturers having several treatment options in their portfolio that can be used in similar treatment groups or interventional areas such as cardiac surgery. The CORCYM Mitral, Aortic aNd Tricuspid Post-maRket Study in a reAl-world Setting (MANTRA) (NCT05002543), sponsored by Corcym S.r.l., is the first clinical trial in the medical device area to adopt the umbrella protocol concept. MANTRA will use a single database to provide post-marketing clinical follow-up data of the entire cardiac surgery portfolio developed by the company for the treatment of heart valve diseases. The common denominator is heart valve disease and the same interventional area (heart valve cardiac surgery) within similar treatment groups, whereas product- and endpoint-focused questions related to the different indications will be addressed in the various substudies and by dedicated analyses of the data obtained through customized case report forms (CRFs).

## Methods

**Fig. 1:**
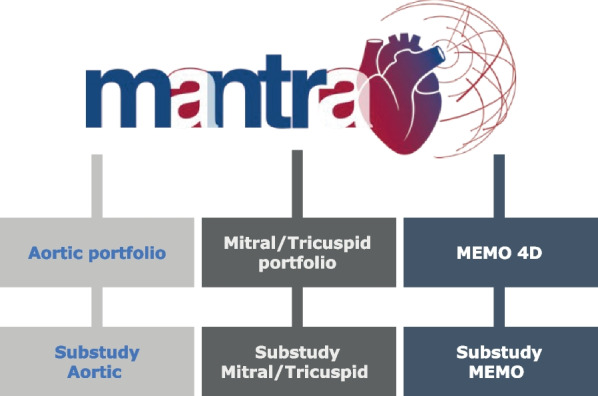
Umbrella structure for the MANTRA study, encompassing heart valve disease

The MANTRA study protocol currently foresees three substudies, outlined in Fig. 1. These substudies target long-term post-marketing safety and device performance data in the treatment areas served by the Corcym product portfolio (manufactured by Corcym S.r.l., Italy, and Corcym Canada Inc.), including data on product accessories such as delivery systems and sizers. Study concept development and data collection have been extensively supported by a Steering Committee consisting of expert cardiac surgeons and cardiologist.

Depending on the Corcym devices used, different institutions can participate in one or more substudies. The study allows for inclusion of isolated as well as combined procedures with multiple device implantation (Sponsor or non-Sponsor devices), with the possibility to follow Corcym safety and performance information on all of the devices implanted and the possibility to collect data on interaction of multiple valve treatments.

The main objective of the aortic and mitral/tricuspid substudies is to monitor the ongoing safety and device performance of the commercially available Corcym products and accessories used for the treatment of aortic and mitral/tricuspid valve disease in a real-world setting, based on standard-of-care data collection. The third substudy focuses on the MEMO 4D device, an advanced technological evolution within the mitral portfolio. This substudy takes advantage of a dedicated echocardiographic CoreLab (Department of Medicine and Surgery, University Milano-Bicocca, Department of Cardiology, Istituto Auxologico Italiano, IRCCS, Milan, Italy) analysis of the product characteristics linked to specific hemodynamic parameters.

### Study design and population

MANTRA is a prospective, multi-arm, global, post-marketing study with long-term follow-up planned through to 10 years. Review and approval by a certified local ethical committee or institutional review board is needed for the master protocol and substudy protocol(s) as per local regulations. All subjects are required to provide informed consent before undergoing any clinical investigation-specific assessments (e.g. quality of life) and treatments.

**Fig. 2:**
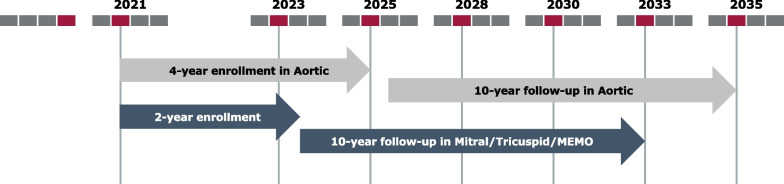
Study timelines study flow from screening to end of study

**Fig. 3:**
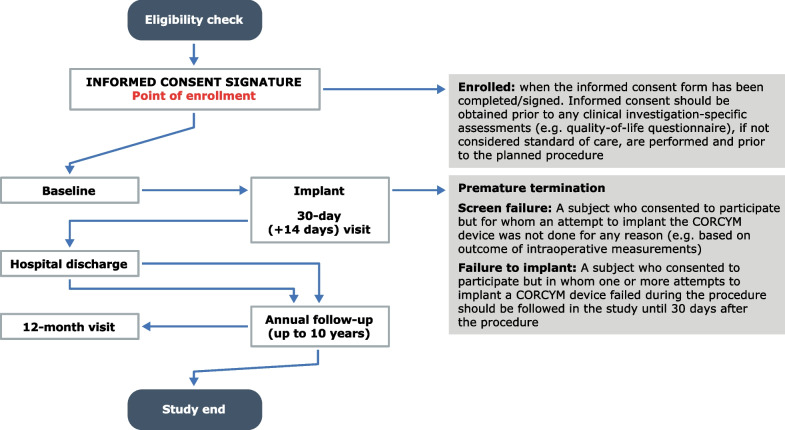
Study flow from screening to end of study

The plan is to enroll approximately 2150 subjects in around 130 sites worldwide over 4 years, (1650 in the aortic substudy, 300 in the mitral/tricuspid and 200 in the MEMO 4D substudy) with follow-up visits scheduled at the time of hospital discharge, at 30 days after the procedure, and then annually up to 10 years (Figs. 2 and 3). Participants diagnosed with heart valve disease who are suitable to undergo valve replacement with a Corcym device per the approved instructions for use and who are willing to comply with the follow-up schedule can be included. Subjects participating in another clinical investigation that could confound the results of the MANTRA study and those with a life expectancy ≤ 12 months will be excluded. Subjects less than 18 years of age, pregnant or breastfeeding women, and other vulnerable populations (e.g. children, or subjects unable to sign informed consent or persons unlikely to be able to comply with follow-up) are not planned to be enrolled. In addition, long-term data obtained from participants treated with either the Perceval Plus™ device or treated with the Bicarbon™ prosthetic valve family and followed under a low international normalized ratio regimen from the SURE-AVR study (NCT02679404), will be transferred into the aortic substudy database.

### Endpoints

The primary endpoint is device success, adapted from the Valve Academic Research Consortium (VARC-3) [3] and from the Mitral Valve Academic Research Consortium (MVARC) guidelines [4] for the aortic substudy and for the mitral/tricuspid and MEMO 4D substudies, respectively. These two guidelines were developed by expert panels in an effort to provide guidance on the appropriate selection of clinical endpoints and definitions to increase consistency in the evaluation and reporting of trial outcomes. Secondary endpoints include mortality, morbidity, hemodynamics, procedural information (e.g. surgical times), intensive care unit management, and patient-related outcomes (e.g. New York Heart Association [NYHA] and quality-of-life improvement). Importantly, the definitions of complications, valve/ring degeneration for safety endpoints are aligned with the current VARC and MVARC guidelines to ensure homogenous reporting, adjudication, and comparison between devices and therapeutic strategies. Furthermore, the study will analyze patient related outcome endpoints, not only related to improvement of the NYHA class, but also considering the improvement of Quality of Life (Kansas City Cardiomyopathy Questionnaire, KCCQ-12) integrated with survival, as suggested by VARC-3 guidelines. Hemodynamic and structural performance from site-reported echocardiography findings, intra-operatively, discharge, at 30 days (+ 14 days), at 12 months after implant and at each subsequent follow-up collected as per the recommendations for the imaging assessment of prosthetic heart valves [5] will be analyzed according to the different device indications. The full list of study endpoints is detailed in Table 2.

**Table 2:** Aortic, Mitral/tricuspid, and MEMO 4D substudy endpoints

	Aortic substudy (n = 1650)	Mitral/tricuspid substudy (n = 300)	MEMO 4D substudy (n = 200)
Primary endpoint	Device success at 30 days (+ 14 days) adapted from VARC-3 guidelines: Technical success Freedom from mortality Freedom from surgery or intervention related to the device or to a major vascular or access-related or cardiac structural complication Intended performance of the valve (mean gradient < 20 mmHg and less than moderate aortic regurgitation)	Device success at 30 days (+ 14 days), adapted from MVARC guidelines: Procedural mortality or stroke; AND Proper placement and positioning of the device; AND Freedom from unplanned surgical or interventional procedures related to the device or access procedure; AND Continued intended safety and performance of the device	Device success at 30 days (+ 14 days), adapted from MVARC guidelines: Procedural mortality or stroke; AND Proper placement and positioning of the device; AND Freedom from unplanned surgical or interventional procedures related to the device or access procedure; AND Continued intended safety and performance of the device
Secondary endpoints	Safety and performance assessments at 30 days, 12 months, and annually after implant Collection of procedural and hospitalization information Patient outcomes	Safety and performance assessments at 30 days, 12 months, and annually after implant Collection of procedural and hospitalization information Patient outcomes	Safety and performance assessments at 30 days, 12 months, and annually after implant Collection of procedural and hospitalization information Patient outcomes

### Schedule of assessments

The study schedule, including assessments and time points, is detailed in Table 3. The schedule follows common standard-of-care practices and may vary across sites. Participants will be asked to complete quality-of-life questionnaires (KCCQ-12 [6] for all the participants and the 5-level EQ-5D version (EQ-5D-5L) [7] for those implanted with the Perceval Plus™ device) at baseline, 30 days, and at 1-year follow-up. Hemodynamic and structural performance (primary and secondary endpoints) will be based on site-reported echocardiographic evaluations for participants enrolled in the aortic and mitral/tricuspid substudies. Echocardiographic examination is planned preoperatively, intraoperatively (pre- and post-implant), at 30 days (+ 14 days) after implantation, at 12 months, and at each subsequent follow-up, if available, as reflected in the standard-of-care at each site. Long-term follow-up will include on-site visits or phone calls.

**Table 3:** Study assessment schedule

Item	Screening/Baseline	Procedure	Hospital discharge	30 Days (+ 14 Days)	12 months (± 30 days)	Annual visits (to 10 years)
Informed consent, eligibility criteria, demographics, medical history, and risk factors, clinical assessment	X					
Vital signs	X		X	X	X	X
Anticoagulant and/or antiplatelet medication	X		X	X	X	X
Blood tests^a^	X^,^		X	X	X	X
Procedural details of implantation and concomitant procedures		X				
Intensive Care Unit (ICU management			X			
Hospitalization data			X			
Pain score assessment (visual analogue scale)			X^b^	X^b^		
Echocardiography^c^	X	X	X	X	X	X
Computed tomography^d^	*X*	*X*	*X*	*X*	*X*	*X*
Electrocardiogram	X		X	X	X	X
NYHA class	X		X	X	X	X
QoL questionnaire^e^	X			X	X	
Serious adverse events		X	X	X	X	X
Device deficiencies		X	X	X	X	X
Accessories deficiencies		X				

^a^White blood cell, red blood cell, hemoglobin, hematocrit, platelets, serum lactate dehydrogenase, haptoglobin, reticulocyte, plasma free hemoglobin, serum creatinine (preoperatively and at discharge visit only), and international normalized ratio/quick. Plasma free hemoglobin will be collected only at sites that have the local capabilities of performing the evaluation. Blood tests baseline ideally be taken on admission, but in any case, not ≥ 7 days before the procedure

^b^Ideally pain score assessment should be collected and entered at post-operative on day 1, day 3, at discharge, and 30 days postoperative

^c^Including collection of images for analysis by Core Lab 3D TEE or 3D TTE images in accordance to standard of care

^d^Computed tomography is optional; only when done as standard of care

^e^KCCQ-12 questionnaire for all the subjects. EQ-5D-5L questionnaire also to be completed by participants implanted with Perceval Plus

For participants enrolled in the MEMO 4D substudy, the echocardiographic parameters reported from the independent echo core laboratory will be analyzed as primary and secondary endpoints. In addition, independent core laboratory evaluation using advanced imaging techniques (intraoperative 3D transesophageal echocardiography and 3D transthoracic echocardiography during follow-up) will be performed to analyze the relationship between the saddle-shaped MEMO 4D annuloplasty ring and the annular motion post-implant and throughout follow-up. Details are described in the echocardiographic protocol.

### Data management and monitoring

Study data will be collected using a web-based electronic data capture system (Merative Clinical Development [formerly IBM Clinical Development EDC system], Ann Arbor, Michigan, USA). Data will be continuously reviewed for omissions, errors, and values requiring further clarification using computerized and manual procedures. Serious adverse events, medical history, and concomitant medications will be coded using standard dictionaries.

Monitoring of the clinical investigation will be an interactive process overseen by the Sponsor monitoring team to ensure that high-quality data is obtained and that the study is conducted in compliance with the protocol, clinical study agreement, applicable laws, regulations, and good clinical practice as established in the Sponsor’s monitoring plan.

The study will be using a risk-based monitoring approach that will include both on-site and centralized monitoring (evaluation without visiting the study site) activities, including full (informed consent and serious adverse events) or partial source data verification, depending on data review findings. Activities of centralized monitoring may include statistical monitoring, contact with the site by telephone, web, or email, examining data quality with programmed data-monitoring checks, and review of adverse events. The intent is to ensure adequate protection of participants enrolled in the study and to ensure the quality and integrity of the study data while focusing resources on the most critical data elements and processes necessary to achieve the study objectives.

### Statistical analysis

Given the non-confirmative nature of the aortic and mitral/tricuspid substudies, neither formal sample size calculations nor formal hypothesis testing will be conducted. In contrast, a sample size calculation was performed for the MEMO 4D substudy, with the endpoint defined as restoration of annular height commissural width ratio (AHCWR) at end-diastole and end-systole at 12 months (± 30 days) after implantation compared with values measured at baseline (improvement versus baseline in AHCWR at post-implant time points indicates that the valve is functioning properly). The sample size calculation was performed only for saddle-shaped size MEMO 4D mitral annuloplasty rings, using the *t-*test for paired means (i.e. before and after device implant) and the following parameters: 90% power, 1-sided 5% significance level, standard deviation of 8 and mean of 5% (assuming a baseline AHCWR of 15% and a postoperative value of 20% at 12 months). AHCWR values range from 20 to 30% when a mitral valve functions properly and from 10 to 17% for diseased valve [8–12]. The sample size calculated using the above parameters was 41 for MEMO 4D featuring a saddle-shape configuration (34−42 mm). The total study sample was 200 and it is projected that at least 60 participants will be treated with MEMO 4D saddle-shape configurated rings, therefore meeting the sample size requirement for the hypothesis testing.

The number and percentage of subjects with device success at 30 days according to the VARC-3 [3] and the MVARC [4] guidelines, along with each individual component, will be presented. According to their statistical distribution, continuous variables will be presented either as means (± standard deviations), minimum and maximum values, or median with interquartile ranges. Categorical variables will be presented as numbers and percentages.

Survival analyses will be conducted to analyze time-to-event variables. Participants without events will be censored at their last known event-free time point. Survival curves will be constructed using Kaplan–Meier estimates; the number of subjects at risk, of censored subjects, of events, and the event-free survival rate and 95% confidence intervals of the survival rate for each time interval will be presented in a summary table.

### Current status of the study

The study was activated in 2021, with the first subject enrolled on July 1, 2021. Forty-four centers have been activated to date, including sites in Europe, North America and Canada, and South Korea. Currently, 393 participants have been enrolled, and follow-up data from 93 participants in the SURE-AVR study have been transferred. Sixty-six of them belong to the Bicarbon™ low international normalized ratio subgroup and twenty-seven to the Perceval Plus™ subgroup. Thirty additional centers are in the process of being activated.

## Discussion

To our knowledge, MANTRA is the first study in the medical device arena to adopt the “umbrella” master-protocol concept. This concept provides a solution to gathering long-term prospective information on the entire Corcym cardiac surgery portfolio products for the treatment of heart valve diseases within a unified trial structure. Accordingly, the original master-protocol concept, developed to provide solutions for complex disease treatments such as oncology, was modified for efficient and economic data collection in the heart valve disease area. There are several advantages to this approach. First, instead of setting up individual studies for each device, a single study program is needed. Second, since many of the participating investigators and sites are using more than one product from the Corcym portfolio and can participate in more than one substudy, a single master-protocol reduces time, resources, and costs for both contract set-up and submissions to ethical committees and institutional review boards. Third, study management and oversight, including data management, monitoring, and data analysis, can be managed by a single team. Having one common database ensure more consistent data return for all the devices with more efficient access for data analysis. Moreover, the data are linked to the company’s quality system, ensuring that valve-related serious adverse events are notified in real time to the vigilance reporting system, ensuring compliance with regulatory requirements in the post-marketing setting.

Further advantages are foreseen for data analysis from the MANTRA study: the Statistical Analysis Plan (SAP) has also adopted the umbrella structure, and a master SAP and substudy SAPs have been created. Having all the data in a single database facilitates the prospective analysis of product families with devices that may be used in more than one indication, with clinical oversight of the experts in the Steering Committee. The structure and broad data output will generate valuable information for submission to regulatory bodies and will support publications and dissemination to the medical community and to the participating institutions, deepening the knowledge on company devices and strengthening the evidence on specific uses. Indeed, the long-term prospective and multicentric information collected in the study, which include safety, performance, and patient related outcomes, will provide clinical evidence to ensure the best treatment option are provided to the patients. On this regard, the data collection in MANTRA includes parameters needed to evaluate the effect of adoption of Enhanced Recovery After Surgery (ERAS) protocols in cardiac surgery. The study can provide information on integration of ERAS to valve surgery (especially in sutureless valve and advanced rings for repair) that could transform it from limited access surgery on to a more “physiological” surgery. This could increase clinical benefits and further improve patient outcomes, especially in high-risk populations needing additional support. We believe that the concept of minimally invasive surgery can evolve even further by going beyond a simple reduction of the surgical incision: by increasing the technical and technological content of the intervention according to a multidisciplinary approach, clinical outcomes after minimally invasive surgery can be enhanced, and postoperative recovery may be fastened with an increased patient and family satisfaction [13, 14].

Furthermore, leveraging on its adaptive design, the MANTRA study may support product development, with the possibility to include ad-hoc substudies and/or study related endpoints to support multiple claims or different indications on old or new products, whenever they become commercially available.

Clearly this concept needs extra efforts in terms of its set-up. Whereas the different treatment areas, albeit all within heart valve disease, show variation in terms of data collection needs and treatment options, they all have to be addressed within the database setup and in the SAP. The Steering Committee has provided extensive input to help refine the data collection. Furthermore, working within a global environment, local expertise is needed to ensure regulatory compliance and diligence, which was solved by contracting with local consultants, who are supporting sites across the globe to obtain approvals and to perform data monitoring. Extra care was taken to review the local national requirements even though the study follows standard-of-care.

Final conclusions on the cost-effectiveness of this approach cannot be drawn at this stage. However, as sites can enroll in one or more substudies, it is assumed that the greatest cost−benefit will be related to start-up costs. For example, if a site participates in all three substudies, start-up costs and ethical review costs apply only once, reducing the total number of administrative procedures that need to be activated. In addition, there should also be a saving in resources, as traditionally these three substudies would have been organized as individual studies, with all study costs multiplied threefold. A further important cost-saver is the costs for development and maintenance of the database. Together, these factors are likely to translate into benefits that will become more evident over time.

## Data Availability

Not applicable.

## Abbreviations

SAP: Statistical analysis plan
CRF: Case report form
VARC: Valve academic research consortium
MVARC: Mitral valve academic research consortium
NYHA: New York heart association
KCCQ: Kansas City cardiomyopathy questionnaire
EQ-5D-5L: 5-Level EQ-5D questionnaire
AHCWR: Annular height commissural width ratio
ERAS: Enhanced recovery after surgery
ICU: Intensive care unit
QoL: Quality of life
TEE: Transesophageal echocardiography
TTE: Transthoracic echocardiography
